# Using road patrol data to identify factors associated with carnivore roadkill counts

**DOI:** 10.7717/peerj.6650

**Published:** 2019-03-29

**Authors:** Samual T. Williams, Wendy Collinson, Claire Patterson-Abrolat, David G. Marneweck, Lourens H. Swanepoel

**Affiliations:** 1 Department of Zoology, University of Venda, Thohoyandou, South Africa; 2 Department of Anthropology, Durham University, Durham, United Kingdom; 3 Institute for Globally Distributed Open Research and Education (IGDORE), Hoedspruit, South Africa; 4 Endangered Wildlife Trust, Johannesburg, South Africa; 5 Eugéne Marais Chair of Wildlife Management, Mammal Research Institute, University of Pretoria, South Africa

**Keywords:** Road ecology, Human-wildlife conflict, Wildlife management, Wildlife-vehicle collision

## Abstract

As the global road network expands, roads pose an emerging threat to wildlife populations. One way in which roads can affect wildlife is wildlife-vehicle collisions, which can be a significant cause of mortality through roadkill. In order to successfully mitigate these problems, it is vital to understand the factors that can explain the distribution of roadkill. Collecting the data required to enable this can be expensive and time consuming, but there is significant potential in partnering with organisations that conduct existing road patrols to obtain the necessary data. We assessed the feasibility of using roadkill data collected daily between 2014 and 2017 by road patrol staff from a private road agency on a 410 km length of the N3 road in South Africa. We modelled the relationship between a set of environmental and anthropogenic variables on the number of roadkill carcasses, using serval (*Leptailurus serval*) as a model species. We recorded 5.24 serval roadkill carcasses/100 km/year. The number of carcasses was related to season, the amount of wetland, and NDVI, but was not related to any of the anthropogenic variables we included. This suggests that roadkill patterns may differ greatly depending on the ecology of species of interest, but targeting mitigation measures where roads pass through wetlands may help to reduce serval roadkill. Partnering with road agencies for data collection offers powerful opportunities to identify factors related to roadkill distribution and reduce the threats posed by roads to wildlife.

## Introduction

Roads, particularly in developing countries like South Africa, are integral to a country’s development and prosperity (Karani, 2008; Keshkamat, Looijen & Zuidgeest, 2009). However, traffic can also have a direct negative impact on both people and wildlife (Verster & Fourie, 2018), with many species at risk from wildlife-vehicle collisions (WVCs), often resulting in an animal’s death, or ‘roadkill’. For the last three decades the field of road ecology has highlighted the negative impacts that roads and their associated users have on biodiversity, and their potential to affect wildlife populations (Kioko et al., 2015; Laurance et al., 2015; Parchizadeh et al., 2018).

Infrastructure development impacts biodiversity and ecosystems through exposing ecological habitats to disturbance and fragmentation (Benítez-López, Alkemade & Verweij, 2010). Land use, land cover, and connectivity within the landscape may change due to expanding road networks (Perz et al., 2013; Liang et al., 2014). Thus a detailed understanding of the factors involved in WVCs (Gunson & Teixeira, 2015) is required to implement successful mitigation strategies. Several studies in the United States of America (USA) and Europe have successfully quantified some of these factors (Gunson & Teixeira, 2015; Rytwinski et al., 2015). As a result, effective mitigation measures have been applied in these areas; habitat connectivity and accessibility are promoted, safe passage for animals using roads is facilitated because natural movements are encouraged and WVCs are reduced (Jackson & Griffin, 2000; Forman et al., 2003; Grilo, Bissonette & Santos-Reis, 2009; Goosem, Izumi & Turton, 2001; Bager & Rosa, 2010).

However, in developing countries, efforts to reduce wildlife mortality around main roads are often hampered due to a lack of research, with other priorities usually dictated by the country’s socio-economic situation (Collinson et al., 2015a). For example, the collection of roadkill data by dedicated research teams can be extremely costly due to the high sampling effort required (Abra et al., 2018). This often limits the number of roadkill studies, and to date only a handful of studies have focussed on roadkill in Africa (Collinson et al., 2015b), and yet these data are vital in order to implement effective mitigation strategies (Gunson & Teixeira, 2015). This is unfortunate because while Africa is incredibly rich in biodiversity (Mittermeier et al., 2011), it also has the fastest growing human population (and associated infrastructure) in the world (United Nations, 2015), which could have serious environmental impacts such as habitat loss, degradation, and fragmentation (Tilman et al., 2017).

A citizen science approach, whereby scientific data are collected by members of the public is one way in which costly sampling strategies can be overcome (Conrad & Hilchey, 2011). For example, as a consequence of citizen science surveys used to monitor the status of birds in the United Kingdom (UK), the UK government introduced targets to reverse population declines identified by the surveys (Gregory & Van Strien, 2010; Greenwood, 2012). While citizen science can be a powerful tool for the collection of scientific data, biases in survey effort can hinder studies of variables such as roadkill rates. An alternative but underutilised source of data on roadkill rates that has the potential to offer more consistent and measurable survey effort, is road patrols. Many highway agencies conduct regular patrols in order to resolve issues that could affect road users. Establishing partnerships between researchers and road patrol agencies could therefore offer significant potential for more effective data collection (Périquet et al., 2018).

In this study, we used data collected by road patrol staff of the N3 Toll Concession (N3TC) to explore the potential for partnering with road agencies to conduct roadkill studies. In 2011, with the aim of managing the impact of roads on wildlife, a partnership was established between the Endangered Wildlife Trust (EWT) and the N3TC, an organisation that operates a 415 km of the N3 highway in South Africa. Following training from the EWT, N3TC patrol staff began collecting data on roadkill incidents. An examination of total roadkill counts showed that the most common carnivore carcass found on the N3 was serval (*Leptailurus serval*). Since this is also one of the more threatened species in the dataset (Thiel, 2015), serval was selected as a suitable indicator species for the study.

The serval is a medium-sized carnivore weighing 8–12 kg (Skinner & Chimimba, 2005). It displays a preference for wetland habitats (Ramesh & Downs, 2015b), the degradation and loss of which constitute the principal threat to serval populations (Thiel, 2011). Although rodents make up a large proportion of serval diets, they are also known to feed on other small prey such as birds, reptiles, and insects (Ramesh & Downs, 2015a; Ramesh & Downs, 2015b). Despite declining numbers throughout their range (Ramesh & Downs, 2013), servals are listed as Least Concern on the IUCN Red List of Threatened Species (Thiel, 2015) and Near Threatened on the South African Red List (Ramesh et al., 2016). Home ranges are estimated to be between 8 and 38 km^2^ (Geertsema, 1985; Ramesh, Kalle & Downs, 2015), and population densities can vary from approximately 6–100 individuals per 100 km^2^ (Ramesh & Downs, 2013; Loock et al., 2018). Servals display largely crepuscular and nocturnal activity patterns (Thiel, 2011; Ramesh & Downs, 2013), but they can also be active during the day (Geertsema, 1985). Although roadkill is thought to present a serious threat to servals (Ramesh et al., 2016), there have been few attempts to quantify these threats or identify associated risk factors.

We used data collected by N3TC road patrol staff as part of routine road patrols to identify which factors were related to serval roadkill counts on the N3. Using a modelling approach, we evaluated the relationships between a set of environmental and anthropogenic variables. Our aim was to determine which variables, if any, were related to serval roadkill counts, and could be used to develop informed management strategies to help mitigate serval roadkill. This approach allowed us to test several hypotheses that could explain serval roadkill patterns (Table 1).

**Table 1:** Hypotheses relating to the relationships between serval roadkill counts and predictor variables included in the models.

Type	Variable name	Variable description	Direction of association	Rationale
Environmental	Season	Wet or dry season	More in dry	Serval may range over larger distances when water is scarce, making them more vulnerable to WVCs
	Wetland	Proportion of 10 km buffer composed of wetland	+	Serval would be more abundant in areas rich in their preferred habitat
	NDVI	Normalized difference vegetation index	+	Areas with a greater NDVI will have greater primary productivity, supporting greater densities of rodents and serval
	Guineafowl	Count of guineafowl carcasses	+	Guineafowl may be preyed upon by serval, increasing serval density
Anthropogenic	Traffic	Average number of vehicles per year	+	More vehicles present more opportunities for WVCs
	Speed	Average speed limit (km/h)	+	Faster cars will make collisions more difficult to avoid
	Road width	Average width of road (m)	+	Wider roads take longer to cross
	Infrastructure	Total number of infrastructure points such as bridges and underpasses	–	Bridges and underpasses may provide more opportunities for serval to cross roads safely

## Materials & Methods

### Study site

**Figure 1:**
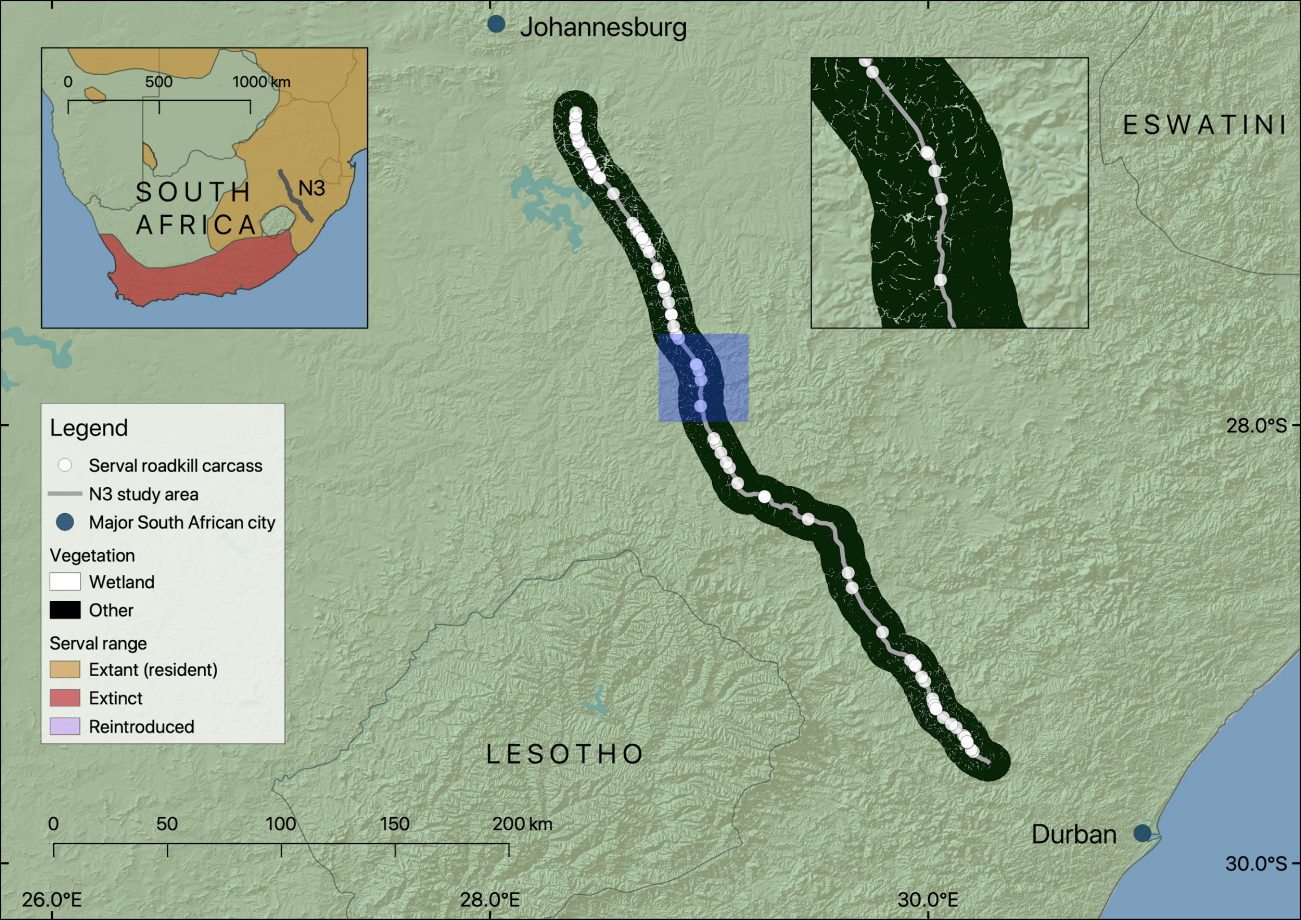
Map showing the location of the section of the N3 studied, serval roadkill carcass locations recorded from 2014 to 2017, and wetland within 10 km of the road. Note that wetland is shown as gaps in the black non-wetland areas within the 10 km buffer. The inset map on the left shows the study area in relation to serval range in South Africa (adapted from Thiel (2015)), and the inset map on the right (location shown in blue) shows a closer view of serval roadkill carcass locations in relation to wetlands.

The study focussed on 410 km of the N3 Toll Route (hereafter referred to as the N3). The N3 is of strategic importance as it links Johannesburg, the country’s largest city, with the port of Durban, and is a major route for the transport of goods between the two cities (Fig. 1). The N3 is classified as a national route, the highest category in the South African road network. Most segments of the national route network are maintained by the South African National Roads Agency (SANRAL), but portions are maintained by provincial, local, or private road authorities. The N3 has been managed by N3TC since 1999 (N3 Toll Concession, 2018). Mean annual traffic volume for a 415 km length of the N3 that encompasses the 410 km stretch studied was approximately 85 million vehicles between 2014 and 2017, and the road is generally two lanes wide in each direction, with speed limits of either 100 or 120 km/h. The N3 passes through three of the eleven South African biomes (Savanna, Grassland and Indian Ocean Coastal Belt (Mucina & Rutherford, 2006)), including urban landscapes, communal land and agricultural areas.

### Data collection

**Figure 2:**
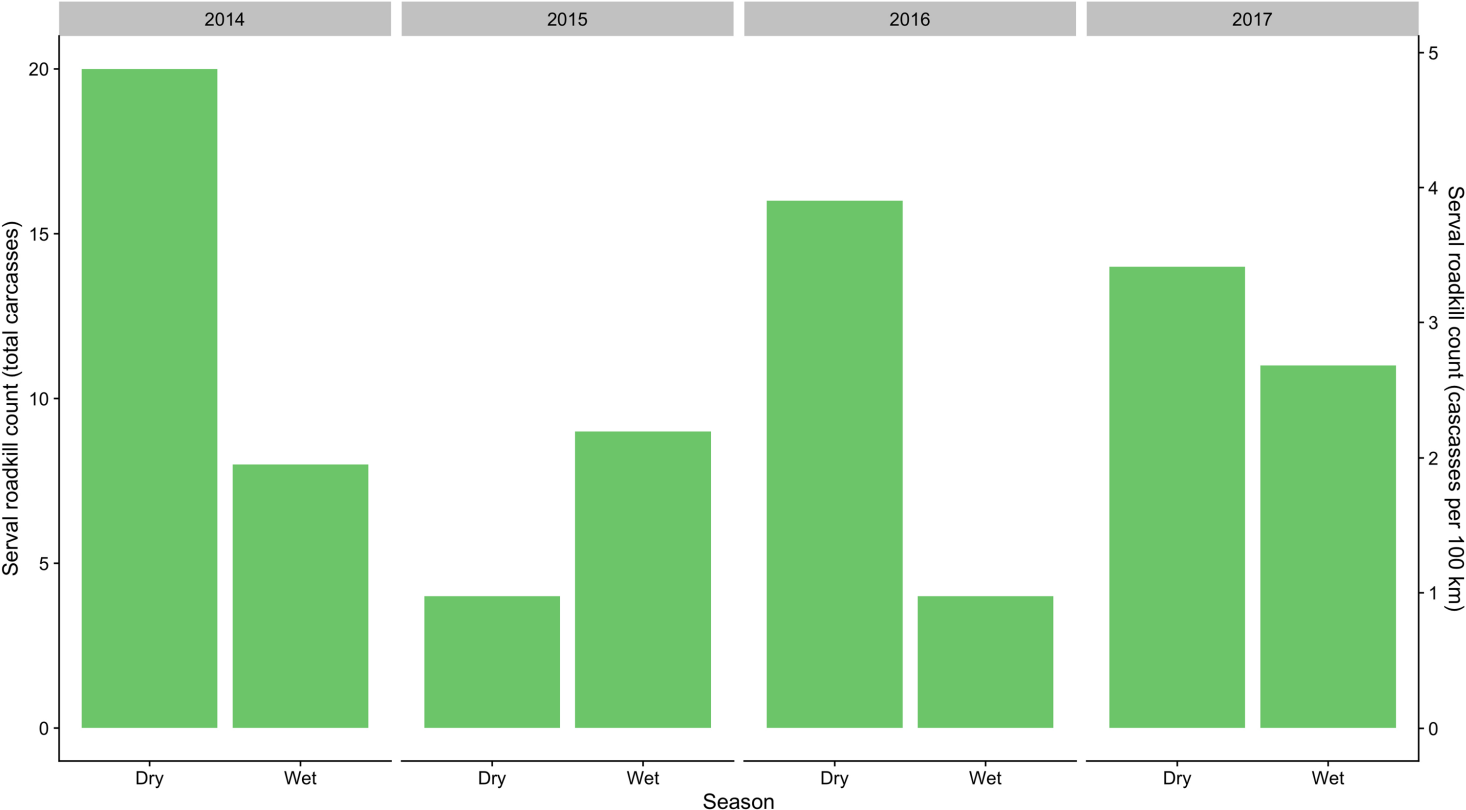
Total seasonal serval roadkill counts collected along a 410 km section of the N3 in South Africa between 2014 and 2017.

N3TC road patrol staff collected field data every day between 01/01/2014 and 31/12/2017 as part of their routine patrol of a 410 km stretch of the N3 between Johannesburg and Durban (Fig. 2).

 The road patrol staff involved in the study undertook annual training with the EWT in wildlife identification and the collection of roadkill data. The day-long training sessions also included training on the importance of conservation, an introduction to road ecology, and how to mitigate the effects of roads on wildlife, in addition to how to follow the data collection protocol. Participants each received a manual and a field guide to identifying animals killed on the road. Staff members from the EWT also accompanied N3TC staff on a sample of their patrols to provide additional training in the field, and to gain a deeper understanding of how the patrols work. Total contact time between the EWT and N3TC staff for training was approximately 14 days per year.

Patrols were conducted four times per day (twice in each direction) at a speed of approximately 100 km/h. Patrol teams mostly consisted of two observers, each allocated six short sections to ensure the entire road was covered effectively. Observers recorded the species of roadkill carcasses encountered, the date and time of the observation, and the carcass location using the nearest route marker, located at 200 m intervals along the road. Carcasses were removed from the road to avoid recounts (Collinson et al., 2014; Guinard, Prodon & Barbraud, 2015). We focussed on analysing records of serval and guineafowl (family Numididae), as these were among the most numerous roadkill carcasses. We classified roadkill observations collected between the 1st of October to the 31st of March as occurring during the wet season, and roadkill collected from the 1st of April to the 31st of September as occurring during the dry season (Cook, Reason & Hewitson, 2004).

We divided the N3 into 41 sampling units, each with a length of 10 km, which corresponded to the home range size of serval (Geertsema, 1985; Bowland, 1990; Ramesh, Kalle & Downs, 2016). We estimated habitat variables by first creating a buffer with a radius of 10 km around each sampling unit, and we used land cover data (Geoterraimage, 2015) to calculate the most common habitat type and the proportion of wetland within the buffer. We also obtained data from N3TC on traffic volumes, speed limits, and the locations of infrastructure such as bridges and underpasses. We measured road width at 410 randomly-generated locations (10 locations per sampling unit) using Copernicus Sentinel satellite imagery (European Space Agency, 2017), measured in QGIS v3.0 (QGIS Development Team, 2018). We used the same locations to sample normalized difference vegetation index (NDVI) provided at a resolution of 250 m every 16 days throughout the study period (Didan, 2015). Road shapefiles were obtained from OpenStreetMap (OpenStreetMap contributors, 2017).

### Data analysis

We conducted all analyses in R v3.5.0 (R Development Core Team, 2018), and all code and data are publicly available (Williams et al., 2018). Within each sampling unit we summed the total number of serval carcasses, which we used as the response variable in our models. All other environmental and anthropogenic variables were also summarised for each sampling unit and used as potential predictors in the models (Table 1).

We modelled the relationship between serval roadkill counts and predictor variables by fitting a generalised linear mixed-effect model using the lme4 package (Bates et al., 2015). Exploratory analyses showed that negative binomial or Poisson distributions were the most plausible for our dataset. The negative binomial distribution with log link outperformed other model structures based on the Akaike Information Criterion (AIC) and visual inspection of a serval roadkill count histogram comparing distributions (Fig. S1), which was expected as the data were over-dispersed with an excess of zeros. This model structure was therefore used for the remaining analyses, following Valdivia et al. (2014).

We included each of the predictor variables listed in Table 1 as fixed terms in the model. To account for spatial autocorrelation we included sampling unit, nested within the most common habitat type, as random terms in the model, including an offset to account for the length of the sampling unit. We checked for patterns in plots of fitted values against residuals to validate the model. Although there may be a slight trend in decreasing residual variation at higher fitted values (Fig. S2), indicating that there could be a more optimal link function, this trend was not excessive and the plot was similar to in other published studies (Valdivia et al., 2014). We also confirmed that there were no patterns evident in plots of predictor variables against residuals. We determined that spatial autocorrelation was not a problem in our models by performing Mantel tests (Mantel, 1967) (Fig. S3) and by inspecting the sample variogram for residuals (Fig. S4). Overdispersion did not appear to be a problem for the final model (θ = 1.08).

## Results

**Figure 3:**
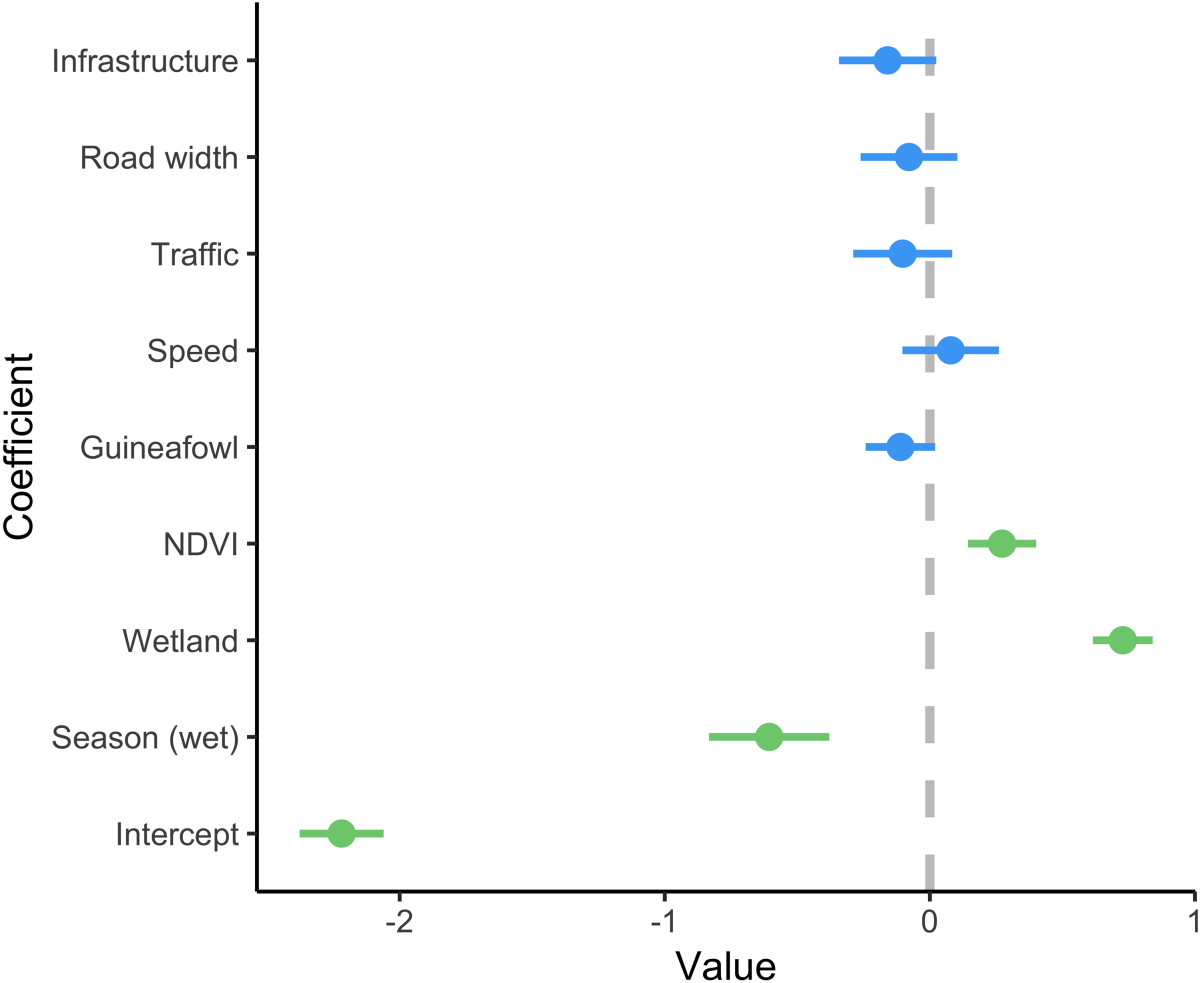
Coefficient estimates showing the effect of predictor variables on serval roadkill counts. Error bars represent 95% confidence intervals. We modelled roadkill counts using a generalized linear mixed effect model with a negative binomial distribution and log link. Coefficients with 95% confidence intervals that overlap zero are shown in blue, and those that do not overlap zero are highlighted in green. The full model summary is provided in Output S5.

**Figure 4:**
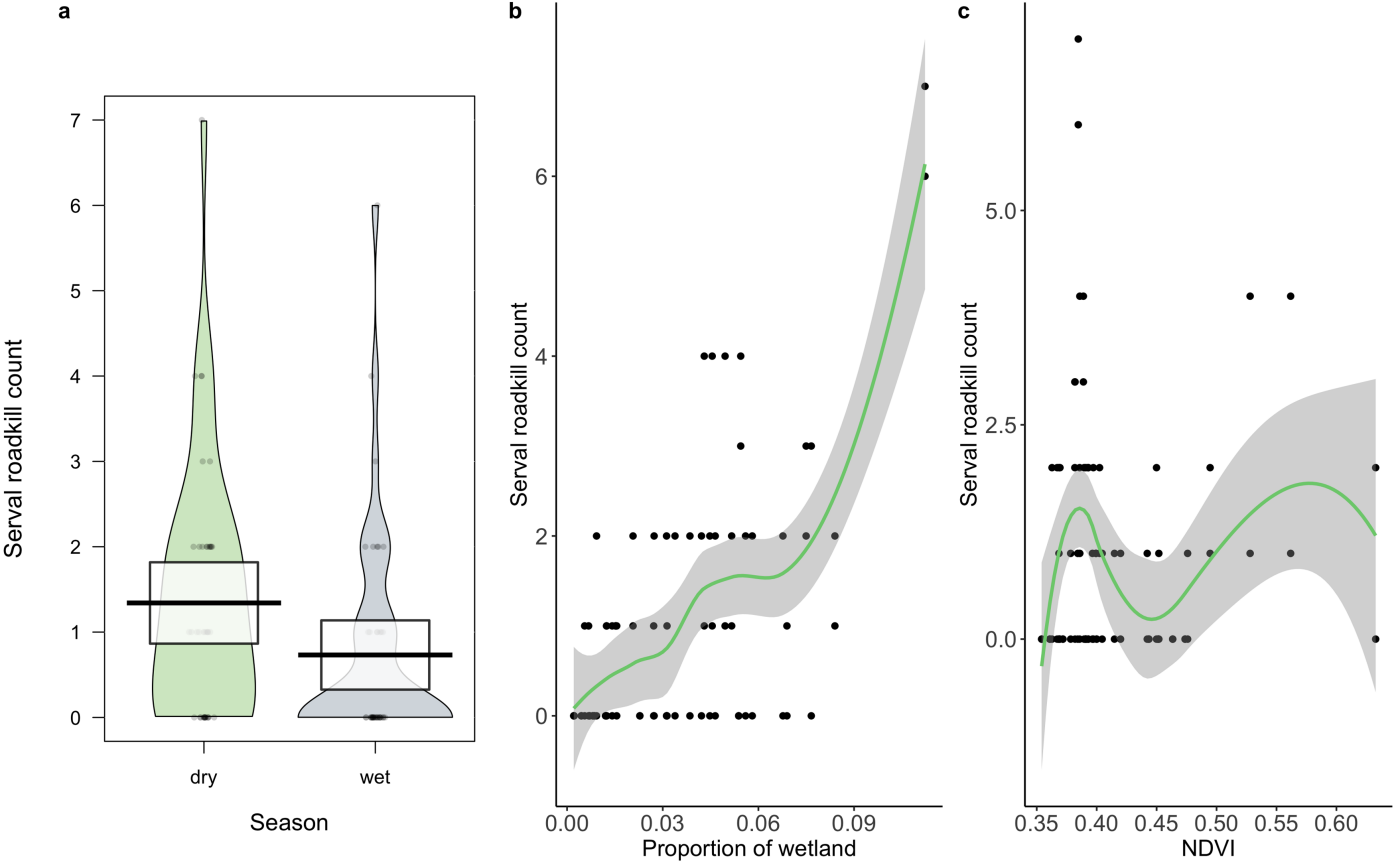
The relationship between serval roadkill counts in each 10 km sampling unit and (A) season, (B) proportion of wetland; and (C) NDVI on the N3 between 2014 and 2017. Boxes (A) and shaded areas (B, C) show 95% confidence intervals.

A total of 86 serval roadkill events were recorded along the study route between 01/01/2014 to 31/12/2017 (Fig. 2), which is equivalent to 5.24 carcasses/100 km/year. Our model showed support for effects of season, wetland, and NDVI on serval roadkill counts (Fig. 3, Output S1). The number of serval roadkills were greater in the dry season than the wet season, and in areas with more wetland, and with greater NDVI (Fig. 4). There was no support for the effects of the number of guineafowl roadkills, or the anthropogenic variables traffic volume, speed limit, road width, or the amount of road infrastructure on serval roadkill (Fig. 3, Output S1).

## Discussion

Conducting twice-daily transects along both lanes of the 410 km study route, solely to collect roadkill count data for research purposes would have been prohibitively costly. Partnering with the road agency facilitated efficient collection of the necessary data in a standardised manner (Collinson et al., 2014). Our findings demonstrate that conservation or wildlife management organisations partnering with road patrol agencies can be a powerful strategy for assessing the various factors associated with roadkill rates. Partnerships could help to efficiently reduce roadkill rates and guide future mitigation measures which would benefit road users and contribute to maintaining biodiversity in a developing landscape (Abra et al., 2018).

We calculated an average of 5.24 serval carcasses/100 km/year (85 carcasses in total) on the N3, but there are few studies with which this can be compared. Serval were among the most common species recorded in a previous study on wildlife roadkill on the N3 (14.8% of 183 citizen scientist records, and 1.5% of 209 road patrol records between 2011 and 2014) (Périquet et al., 2018). Five of 17 collared serval were killed by snaring, hunting dogs, and WVCs in the Drakensberg Midlands, South Africa (Ramesh, Kalle & Downs, 2016), but the breakdown of these mortality sources is unclear. Serval roadkill has also been occasionally recorded elsewhere in Africa, with two serval carcasses recorded in a study on roadkill in Uganda (Cibot et al., 2015), but without data on survey effort this cannot be converted into a rate for comparison.

The rate of serval roadkill on the N3 is roughly in line with roadkill rates of other carnivores such as spotted hyaena (*Crocuta crocuta*), common genet (*Genetta genetta*), and black-backed jackal (*Canis mesomelas*) in the Tarangire–Manyara ecosystem, Tanzania (each at approximately 5 individuals/100km/year) (Kioko et al., 2015). Carnivore roadkill rates in Portugal were also similar (5–6 individuals/100 km/year) for species including Egyptian mongoose (*Herpestes ichneumon*), Eurasian badger (*Meles meles*), and common genet (Grilo, Bissonette & Santos-Reis, 2009). However, carnivore roadkill rates can vary considerably in relation to factors such as species behaviour, abundance, and road placement, for example from 1 to 20 individuals/100 km/year for Western polecats (*Mustela putorius*) and red fox (*Vulpes vulpes*) respectively (Grilo, Bissonette & Santos-Reis, 2009).

We found support for the hypothesis that serval roadkill counts would be higher in the dry season than the wet season, as carnivores tend to have larger home ranges when water is scarce (Macdonald & Loveridge, 2010), which could put them at greater risk of WVCs. We also found support for the hypothesis that serval roadkill counts would be positively related to the amount of wetland, which suggests that servals are being killed on roads located in their preferred habitat (Ramesh & Downs, 2015b), probably because they are more abundant in these areas (D’Amico et al., 2015). Similar findings were found with the raccoon dog (*Nyctereutes procyonoides viverrinus*) in Japan (Saeki & Macdonald, 2004), spotted turtles (*Clemmys guttata*) and Blanding’s turtles (*Emydoidea blandingii*) in North America (Beaudry, deMaynadier & Hunter, 2008), and owls in Portugal (Gomes et al., 2009). Finally, our findings support the hypothesis that there is a positive association between NDVI and roadkill counts of serval. Areas with greater NDVI tend to have higher levels of primary productivity (Tucker et al., 1985).

There may be a weak relationship between serval roadkill counts and some predictor variables such as infrastructure, and with further data collection this may become more evident. Nevertheless using the current dataset our model did not show support for our other hypotheses, including that serval roadkill counts would be associated with guineafowl roadkill counts, or with anthropogenic variables such as traffic volume, speed limit, road width, or the amount of road infrastructure. This suggests that serval roadkill counts are influenced primarily by ecological drivers such as season, wetland, and NDVI, and that this will vary between species with different ecological requirements. As a result roadkill mitigation strategies may need to be tailored to target species rather than relying on a more generalised approach.

Without data on serval population sizes or other sources of mortality, it is difficult to determine how important WVCs may be for serval population dynamics along the study route. Our findings nevertheless appear to support the suggestion by Ramesh et al. (2016) that WVCs could be a major source of mortality for the species, which is also true for other carnivores. Over 40% of Eurasian badgers in southwest England, for example, were thought to be killed each year in WVCs (Clarke, White & Harris, 1998). WVCs accounted for 35% of annual mortality of Florida panthers (*Puma concolor coryi*) in the USA (Taylor et al., 2002), 4–33% of annual mortality of jaguars (*Panthera onca*) in the Atlantic Forest, Brazil (Cullen Jr et al., 2016), and 17% of annual mortality of Iberian lynx (*Felis pardina*) in southern Spain (Ferreras et al., 1992). Identifying factors associated with high roadkill rates is a useful first step in developing roadkill mitigation strategies that could contribute significantly to conservation efforts of some species. Findings from this study suggest that serval roadkills could be reduced if mitigation efforts are focussed on wetland areas and immediate surrounds.

Installation of fencing in combination with wildlife crossing structures (Clevenger, Chruszcz & Gunson, 2001) like under- and over-passes have been used extensively in Europe and America (Danielson & Hubbard, 1998; Goosem et al., 2008; Bissonette & Rosa, 2012), and could be applied to a South African context provided the tendency of serval to use these structures is adequately assessed before implementation. Wildlife crossing structures are widely used by many species (Clevenger, Chruszcz & Gunson, 2001) including carnivores (Grilo, Bissonette & Santos-Reis, 2008; Andis, Huijser & Broberg, 2017), and it is reasonable to infer that serval would make use of them as well. The movement patterns of the target species need to be clearly understood, as these are often associated with drainage lines, topography, and habitat (Clevenger, Chruszcz & Gunson, 2001; Rytwinski et al., 2016). Correct tunnel design at sites optimal for the target species need to be well thought out, as does the installation of the tunnels (Danielson & Hubbard, 1998). The use of fences must also be carefully considered prior to their use. Fences that are too short in length may exacerbate the problem (Seiler, 2005) by causing wildlife to follow the fence until the end is reached and there is a gap to cross, thus creating a fence-end hotspot (Seiler, 2005). Many animals will also either dig under, push through, or jump over fences and consequently collide with vehicles (Van Niekerk & Eloff, 2005). Caution should be used in applying this approach, because while fencing can be effective at mitigating WVCs, it can also disrupt migration and dispersal routes, reduce gene flow, and exacerbate ecosystem fragmentation (Creel et al., 2013), while providing materials that can be used by poachers to construct snares (Lindsey et al., 2011; Williams et al., 2016). The benefits of using of fencing must therefore be carefully weighed against the costs.

Previous research suggests that using the above approach in combination with other methods such as implementing low speed zones, installing traffic-calming devices such as speed bumps (Glista, DeVault & DeWoody, 2009) or using species-specific warning signage placed in strategic locations to alert road users (Hardy, Lee & Al-Kaisy, 2006) may increase the effectiveness of mitigation measures (Grace, Smith & Noss, 2015; Collinson, Marneweck & Davies-Mostert, 2019). While the standard static warning signage typically used is often largely ineffective since drivers quickly habituate to it and fail to make adequate reductions in speed (Huijser et al., 2015), recent research has shown that when optimised warning signs can be successful at changing driver behaviour and reducing WVCs (Collinson, Marneweck & Davies-Mostert, 2019). Enhanced warning signs that are specific in time and space may also be effective (Huijser et al., 2015). While it is possible to modify driver behaviour, our data suggest that the anthropogenic variables included in our models may not be as significant as the various environmental variables in affecting serval roadkill rates on the N3. Removal of roadside vegetation that supports abundant rodent species (Ruiz-Capillas, Mata & Malo, 2015) could reduce the rate of serval roadkill, although this could impact plant and small mammal conservation. Keeping grass verges trimmed rather than removing vegetation entirely may be effective in manipulating the landscape of fear (Jacob & Brown, 2000) causing wildlife to spend less time in verge habitats without altering their abundance. If grain spillage from agricultural or transport vehicles is a common occurrence (Ansara, 2008; Gangadharan et al., 2017), the increased food availability could boost rodent abundance, drawing predators into the road and leading to increased WVCs (Gangadharan et al., 2017), and this is therefore another factor that needs to be investigated before developing mitigation strategies.

Although useful, there were some caveats to our approach. For example, road patrol teams did not collect data that would have facilitated modelling the number of missed detections (Shilling, Perkins & Collinson, 2015). Furthermore, recording data on which patrol teams conducted which patrols would have enabled us to control for inter-observer bias (Shilling, Perkins & Collinson, 2015), and is recommended for future studies. We also created a WhatsApp group to help observers receive rapid responses to future queries about species identification or any other questions, which we hope will help ensure accurate data collection. We were unable to record the age-sex class of carcasses, which would have been interesting to include in the models (Pallares et al., 2015). We recommend collection of tissue samples to allow this in future studies. Additional data such as the density of serval and prey species at different sampling points along the length of the road would have been incredibly useful in determining the impact on local populations. Finally, the speed at which patrols were driven could bias roadkill detection towards larger species (Collinson et al., 2014), so this method would not be suitable to collect roadkill counts for all species. Further studies incorporating missed carcass detections, inter-observer bias, and population density of target species would be worthwhile. Despite these limitations, this study demonstrated that even the collection of very limited data such as the location, date, and species (or taxon) of roadkill carcasses can help to inform wildlife management policy while minimising additional workload burden on road patrol staff.

## Conclusions

Integrating roadkill data collection into existing road patrols can provide an efficient means of collecting data to allow identification of factors associated with carnivore roadkill. Our findings support the hypotheses that serval roadkill counts were higher in the dry season than the wet season, and they were also higher in areas with more wetland and in areas with greater NDVI. Anthropogenic factors such as traffic volume, speed limit, and the amount of road infrastructure did not influence serval roadkill counts. We suggest that efforts to mitigate serval roadkill, such as installing wildlife crossing structures in combination with fencing, should be targeted at wetlands, but this must be tailored to the ecological requirements of target species.

##  Supplemental Information

10.7717/peerj.6650/supp-1 Figure S1 Histogram showing distribution of serval roadkill counts in relation to a range of distributions

10.7717/peerj.6650/supp-2 Figure S2 Scatter plot of fitted vs residual values for the full generalized linear mixed model with negative binomial distribution

10.7717/peerj.6650/supp-3 Figure S3 Mantel tests for the full generalized linear mixed model with negative binomial distribution

10.7717/peerj.6650/supp-4 Figure S4 Variogram from for residuals for the full generalized linear mixed model with negative binomial distribution

10.7717/peerj.6650/supp-5 Supplemental Information 1 Summary of full generalized linear mixed model with negative binomial distribution

## Additional Information and Declarations

### Data Availability

The following information was supplied regarding data availability: The dataset is available at Figshare: Williams, Sam; Collinson, Wendy; Patterson-Abrolat, Claire; Marneweck, David G.; H. Swanepoel, Lourens (2019): Serval roadkill dataset. Figshare. https://doi.org/10.6084/m9.figshare.6833378.v1.
