# Supplementary figures and images for: Using road patrol data to identify factors associated with carnivore roadkill counts
Source: PeerJ. 2019 Mar 29;7:e6650. doi: 10.7717/peerj.6650 (PMC6445248; doi:10.7717/peerj.6650)

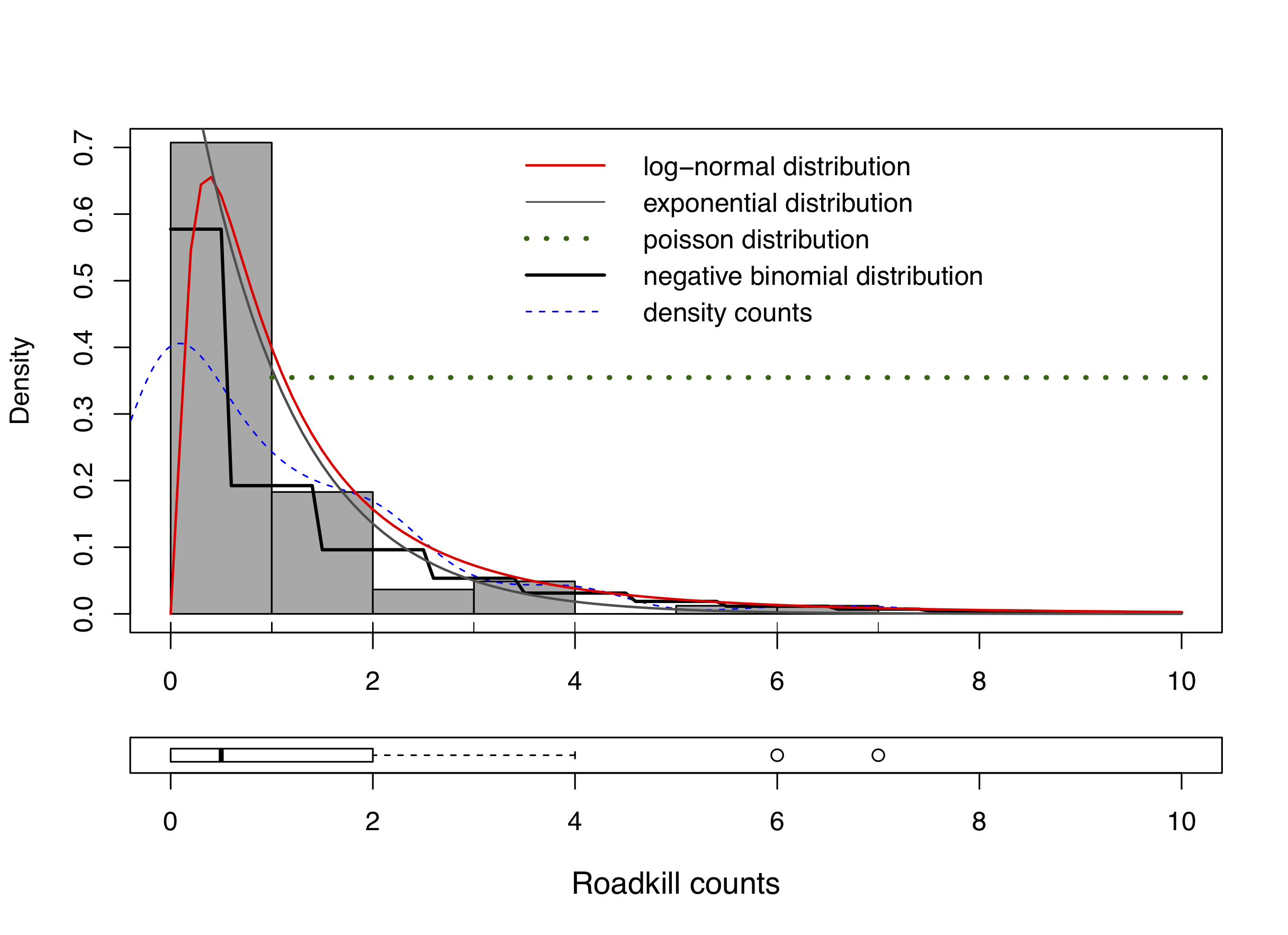

Supplement: Figure S1 [file peerj-07-6650-s001.png]

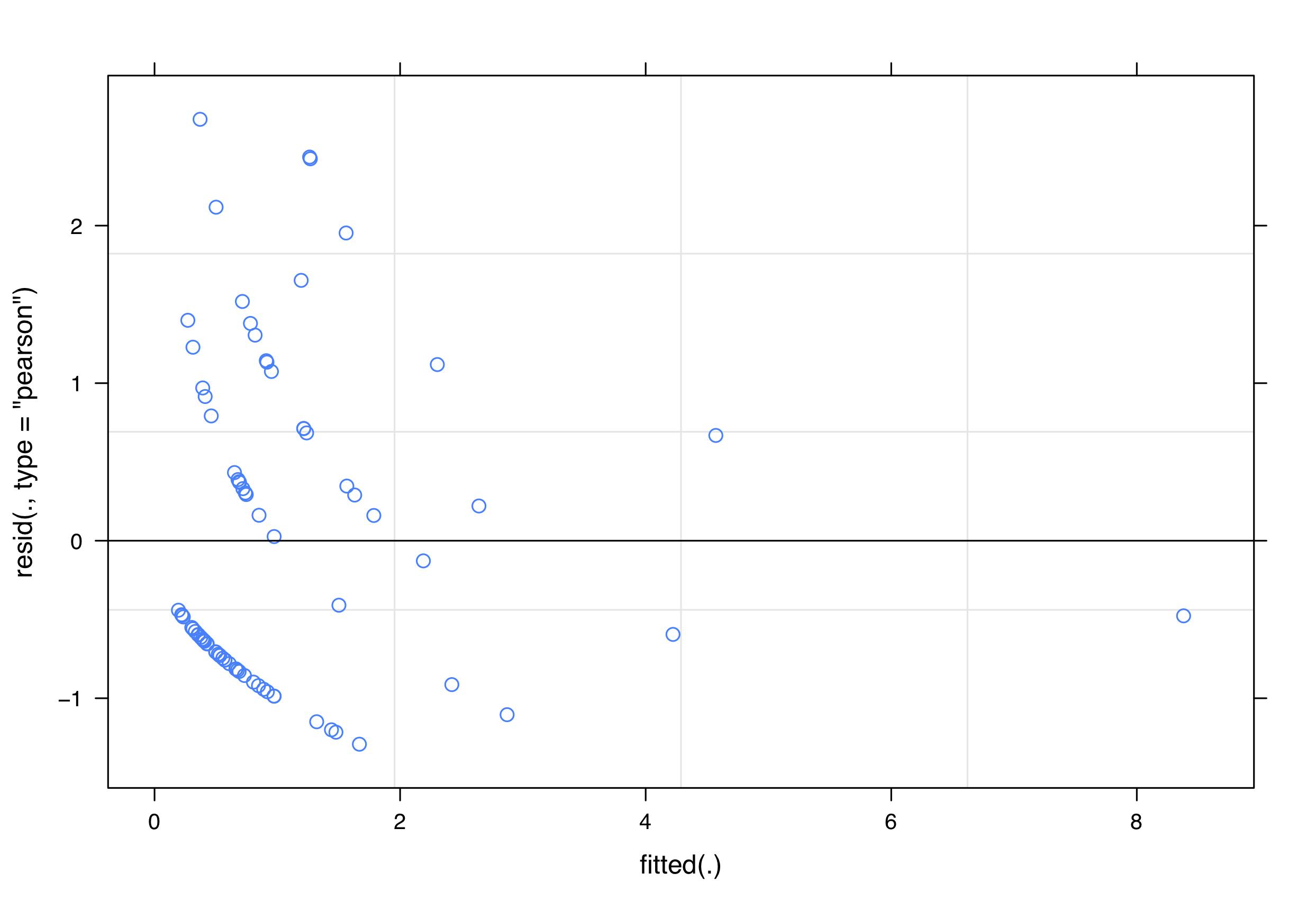

Supplement: Figure S2 [file peerj-07-6650-s002.png]

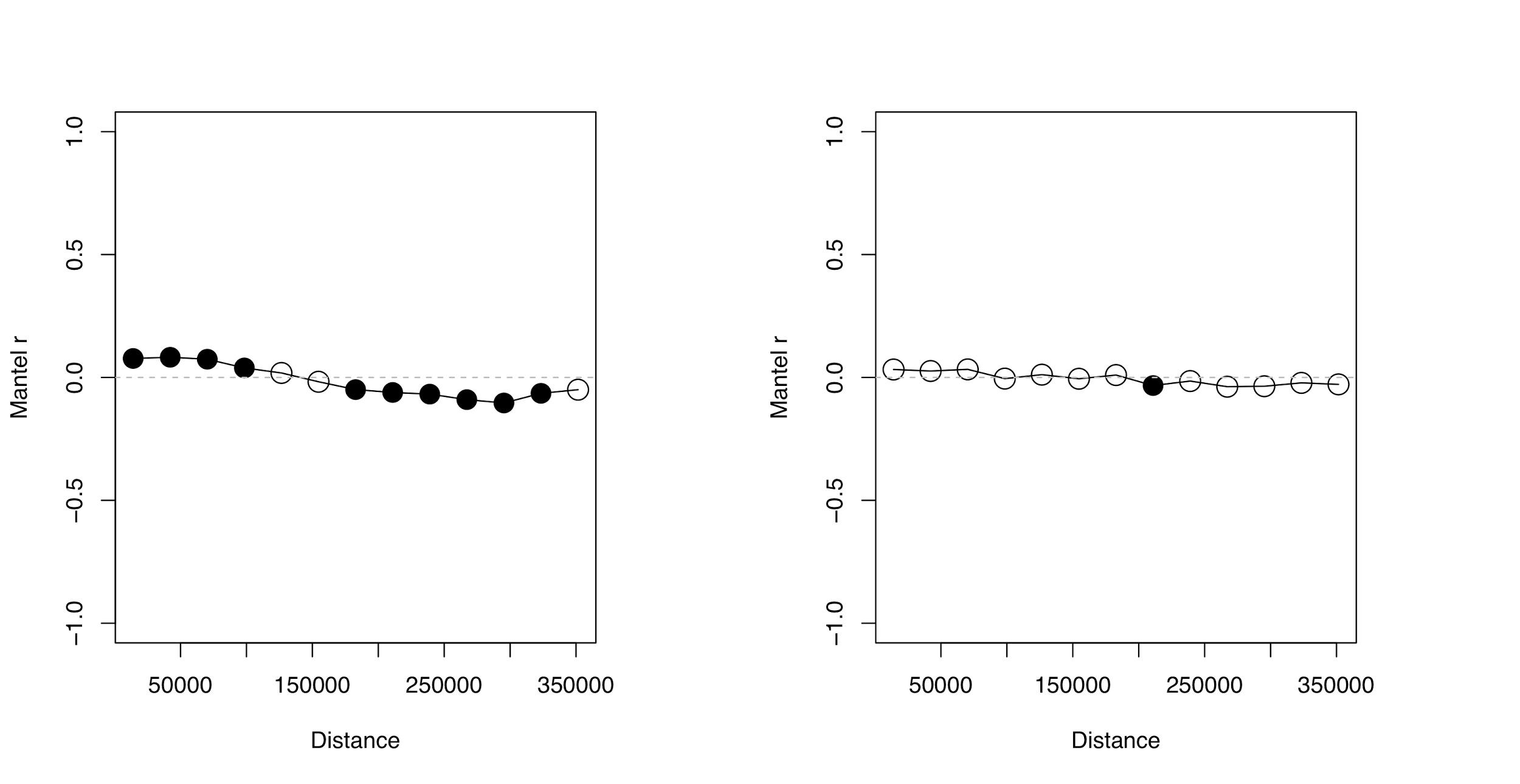

Supplement: Figure S3 [file peerj-07-6650-s003.png]

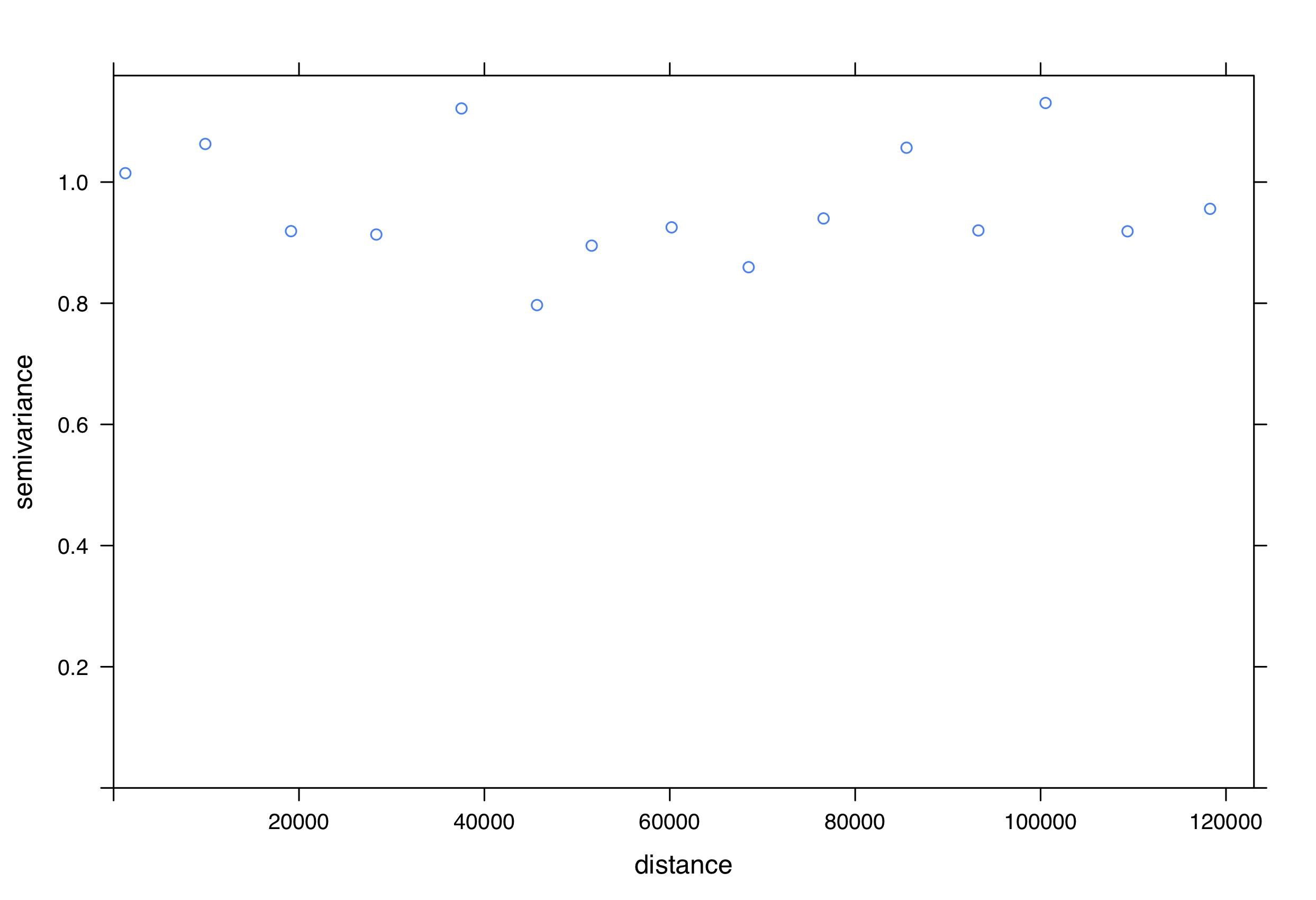

Supplement: Figure S4 [file peerj-07-6650-s004.png]
